# The “Rotten” matter in A Farewell to Arms: An Ecological Gothic reading

**DOI:** 10.12688/f1000research.75482.2

**Published:** 2022-11-23

**Authors:** Lay Sion Ng

**Affiliations:** 1 CEGLOC, Faculty of Humanities and Social Sciences, University of Tsukuba, Tsukuba, Ibaraki, 305-8577, Japan

**Keywords:** eco-gothic, non-anthropocentrism, rotten food, deformed bodies, gothic rain, broken landscape, eco-resistance, anthropocentric warfare

## Abstract

This article uncovers the gothic tropes manifest in the “rotten” food, human bodies, landscapes, and rain in Ernest Hemingway’s A Farewell to Arms through an eco-gothic perspective. It demonstrates how the rotten food, the disjointed bodies, the broken landscapes, and the gothic rain can be viewed in the novel as counter-narratives against the narratives of war, the military, and modern medicine. The first part of this article suggests interpreting war as a form of cannibalism by exploring the representations of rotten food and the connection between eating and killing. Next, the author focuses on how the body is fragmented both metaphorically and literally by the discourse of war, the military, and medical science. The third part uncovers the non-anthropocentric consciousness embedded within the protagonist’s narrative, followed by the gothicizing and romanticization of nature in the fourth section. Here, the protagonist’s linking of the human body to the natural landscape, the descriptions of the gothic rain, and the romanticized snow—all these, as the author argues, can be interpreted as a collective resistance against industrial, anthropocentric warfare.

## Introduction

Ernest Hemingway’s *A Farewell to Arms* depicts deformed and fragmented bodies, matters, and landscapes. In the novel, these aspects are embedded within the word “rotten,” which is used to depict the awful condition of the battlefields and war ( *AFTA,* 29), the food (46), the combat life (63), romance (26), and luck (49). According to *Oxford Learners’ Dictionaries*, the word “rotten” is defined as “things—such as food or wood—that have decayed and cannot be eaten or used.” *Macmillan Dictionary* defines it as “terrible,” “unpleasant,” “looking, or feeling ill.” These implications—decaying, terrible, ill, unpleasant—embedded in the word “rotten” thus indicate a certain uncanny ^1^ and grotesque ^2^ quality. This article uncovers the gothic tropes manifest in *rotten* food, human bodies, landscapes, and rain in *A Farewell to Arms.* From an ecological gothic perspective, these elements serve as counter-narratives to the narratives of war, military, and modern medicine, making the egocentric delusion of the Great War visible.

M. H. Abrams defines “gothic” as an “atmosphere of gloom and terror” that “represents events that are uncanny or macabre or melodramatically violent” (qtd. in Silviya and Immanuel, 25). Ecological Gothic is a relatively new field that merges two traditionally dissimilar fields, one with a biocentric focus and one that focuses on “humanity” and “human desires, fear, and trauma” (Deininger and Cox, 166). ^3^ In his article, David Del Principe states, “the EcoGothic examines the construction of the Gothic body—unhuman, nonhuman, transhuman, posthuman, or hybrid—through a more inclusive lens, asking how it can be more meaningfully understood as a site of articulation for environmental and species identity” (1). The Ecological Gothic is thus a framework attempting to destabilize anthropocentrism by blurring or challenging the hierarchical boundaries between humans and nonhumans, the mind and the body, the self and the other.

The connection between Hemingway and the ecological gothic can be traced back to the author’s ambiguous relationship with nature. As Terry Tempest Williams notes, Hemingway’s relation to the natural world is shown in opposition: “hunting and loving. Physical and spiritual. Life and death. Controlling masculine and wild feminine” (Grimes, 104). This multidimensional perception of the natural world creates a possibility for an ecological gothic reading of his work. Moreover, the author’s high interest in “materiality” and sensitivity to “place-energies” are following the material ecocritical approach ( Godfrey, 2016: 94, 95). Hemingway’s consciousness of the force, energy, and agency possessed by materials in places as well as sites themselves are shown in Williams’ description of him as “a powerful mentor, in terms of what it means to create a landscape impressionistically on the page, to make it come alive, pulse, breathe” (qtd. in Godfrey, 2006: 48). ^4^

## Research methodology

In this article, I utilize diverse analyses concerning the field of material ecocriticism, ecological gothic, the environmental history of The Great War, the previous ecocritical readings of Hemingway’s *A Farewell to Arms*, and biographical studies of Hemingway. More specifically, Andrew Smith and William Hughes’ edited collection *EcoGothic* (2013) and Serenella Iovino and Serpil Oppermann’s edited collection *Material Ecocritism* (2014) serve as the primary research methodology. Secondary literature used include David Del Principe’s “The EcoGothic in the Long Nineteenth Century” (2014), William Hughes, David Punter, and Andrew Smith’s edited book named *The Encyclopedia of the Gothic* (2015), Maria Concetta Dentoni’s “Food and Nutrition (Italy)” in *International Encyclopedia of the First World War*, Joseph P. Hupy’s “The Environmental Footprint of War” ( 2008), to name a few. Analyzing Hemingway’s work from the lens of eco-gothic is a relatively new thing. However, some previous studies help construct the arguments in this article. This includes Trevor Dodman’s “‘Going All to Pieces’: *A Farewell to Arms* as Traumatic Narrative” ( 2006), Gruber Laura Godfrey’s *Hemingway Geographies* ( 2016), David A. Rennie’s “The Real British Red Cross and Hemingway’s *A Farewell to Arms*” (2018), and so forth.

This article is divided into four sections. The first section examines the representations of rotten food as opposed to clean and fresh food in the novel. Here, the act of eating is juxtaposed with that of killing, leading to the argument that war is a grotesque form of cannibalism. Secondly, I concentrate on how the body is fragmented metaphorically and literally by the discourse of war, the military, and medical science. What does such fragmentation say about bodily boundaries and the integrity of the self? These questions are pondered in this section. The third part uncovers the non-anthropocentric consciousness embedded within Frederic’s narrative. Here, I see Frederic’s linking of the human body and the natural landscape as a form of eco-resistance against industrial warfare. Finally, I explore Frederic’s gothicizing and romanticization of nature in the novel and suggest that the gothicized rain/snow and romanticized rain/wind can also be seen as a counter-narrative to the anthropocentric war.

## The rotten food

### “I was blown up while we were eating cheese”

The rotten food in the novel serves as a *storied* ^5^ matter that makes the war’s monstrosity visible. In the novel, the most representative rotten-food episode is in chapter nine. Prior to Frederic’s injury and Passini’s death, the ambulance team is having some “rotten” macaroni, cheese, and wine at the front ( *AFTA,* 46). Here, the awful condition of the macaroni and wine (“It tasted of rusty metal” (46)), the dirty cheese (“its smooth surface covered with brick dust” (46)), the lack of any utensils (“I put thumbs and fingers into the macaroni and lifted” (46)), and the awkwardness of eating the macaroni (“They were all eating, holding their chins close over the basin, tipping their heads back, sucking in the ends” (46)) make a mockery of the ideal heroism of soldiers and the absurd glamor of war.

Frederic’s linking of food to the war and its absurdity can be found in his description that “I had seen nothing sacred, and the things that were glorious had no glory and the sacrifices were like the stockyards at Chicago if nothing was done with the meat except to bury it” ( *AFTA*, 161). To Frederic, the meaninglessness of human sacrifice is derived from the absurdity of war itself. Indeed, several instances show that warfare is absurd. For example, the military department decides to decorate Frederic with a “silver medal” (105) even though he has done nothing heroic during the fight: “I was blown up while we were eating cheese” (55). The military department does this to enhance its image for civilians; on seeing his medal, one may imagine that Frederic, who represents the Italian army, had “killed two hundred Austrians or captured a whole trench” during the fight (105). The system of military decorations is, therefore, a fraud. Another episode that shows the war’s absurdity is during the Caporetto retreat when the Italian military police begin to fire on their own people suspecting, in their fear, that the Germans have donned Italian uniforms to infiltrate themselves among the Italian soldiers. Aymo, a comrade of Frederic, is shot to death due to this. Moreover, to hide their own mistake, those frightened Italian battle police will have to kill more of their own people: “They’d have to shoot us all to prove they were right the first time” (185). Acknowledging these absurd incidents, Frederic concludes,

There were many words that you could not hear and stand to hear and finally only the names of places had dignity … Abstract words such as glory, honor, courage, or hallow were obscene beside the concrete names of villages, the numbers of roads, the names of rivers, the numbers of regiments and the dates.

( *AFTA,* 161)

In addition to the military reward system, the food distribution system in the military is also problematic. This aspect is revealed in the conversation between Frederic and Gino before the Caporetto retreat. According to Gino, there is an issue regarding the distribution of food: “The regiments in the line get pretty good food but those in support don’t get so much. Something is wrong somewhere. There should be plenty of food” ( *AFTA,* 160-61). “The dogfish are selling it somewhere else,” Frederic then concludes (161). Agreeing with Frederic, Gino states, “[i] t is very bad for the soldiers to be short of food. Have you ever noticed the difference it makes in the way you think?” (161). While Frederic’s statement confirms the corrupted food distribution system as *rotten*, Gino’s description foreshadows the important role food plays in times of war. In other words, insufficient food distribution leads to starvation, representing a form of physical and mental violence—among the soldiers, which ultimately results in defeat in war. Historically, after the Caporetto retreat, Silvio Crespi, the head of the Commission for Procurement and Consumption in the Italian army, decided to change the soldiers’ diet as he believed that the retreat was caused by the soldiers’ poor nutrition ( Dentoni, 2014). From this respect, the *rottenness* of the food in the novel underscores the absurdity of war and the corruption of military systems during the era of World War One.

### “We are all cooked”

In the novel, the vicious quality of the war is further emphasized in Rinaldi’s symbolic joke in Chapter Twenty-Five. During dinner time, Rinaldi sarcastically jokes that the meat-stew they are eating is the body of a “dead Austrian” (152). Here, the act of eating cooked meat is juxtaposed with the act of killing living humans, leading one to see warfare as a form of cannibalism (and hence, barbaric). It is under this implication that the food is claimed as *rotten* since barbarity has become the main component of it.

Another symbolic instance of cannibalism is reflected in Frederic’s repetition of the phrase “am cooked” seven times in chapter twenty-one. According to the *Farlex Dictionary of Idioms*, the term means physically ruined, mentally depressed, and emotionally traumatized. In his conversation with Rinaldi, Frederic claims, “[w] e are all cooked. The thing was not to recognized it. The last country to realize they were cooked would win the war” (116). Following this, the key to winning the war is to repress the trauma suffered by a nation because of the war. That is to say, to win the war for one’s country, one must *cook* (traumatize) oneself by *cooking* (killing) the other and then pretend that one is not yet *cooked* (ruined). Indeed, this method is beyond human limits. As Rinaldi reveals, “this war is killing me … I am very depressed by it” (146). War is, therefore, a *slow-cook* process that transforms one into both the eater and the eaten. It is in this sense that warfare can be seen as cannibalistic and hence, monstrous.

## The *rotten* bodies

### “It was his knee all right. The other knee was mine”

Besides the rotten food, the wounded body represents another agentic matter that underlines the issue of war’s rottenness. In this case, it is necessary to explore the incident of Frederic Henry’s knee injury. Frederic gets injured when one of the Austrian’s trench mortar shells hits him during their mission to send four ambulances up to the river. When Frederic realizes that his comrade, Passini, is already dead and that the mortar shell has hit him on his knee, Frederic panics: “I looked at my leg and was very afraid”; “My knee wasn’t there. My hand went in, and my knee was down on my shin” ( *AFTA,* 48). The uncanny out-of-body experience further enhances the panic Frederic had a moment ago: “I felt myself rush bodily out of myself and out and out and out and all the time bodily in the wind … I knew I was dead … Then I floated, and instead of going on I felt myself slide back” (47). Here, Frederic’s helpless condition—his out-of-body experience and witnessing Passini’s death—makes him realize that he has no control over his own body and possible death. Moreover, Frederic’s experience of derealization/depersonalization ^6^ leads him to suffer from post-traumatic stress disorder (PTSD) in the latter part of the story.

Frederic’s PTSD symptoms ^7^ can be found in several aspects. For instance, he suffers from sleeping problems and a disturbing nightmare: “I slept heavily except once I woke sweating and scared and then went back to sleep trying to stay outside of my dream” ( *AFTA,* 77). He also suffers from excessive drinking, causing him to have “jaundice” (124) and attempts to numb his emotions as a way to avoid being reminded of the traumatic event: “The head was mine, but not to use, not to think with … [and] not too much remember” (199). This leads him to depression.

Frederic’s rejection of his wounded knee, his claiming its ownership to the doctor— “It was his knee all right. The other knee was mine” (199)—comes from his psychological disgust towards its foreignness. He begins to perceive the wounded knee as a grotesque Other after it is scanned by an X-ray machine and seen by the doctors. Through the “eye” of the device, Frederic notices that some “foreign bodies” have “invaded” and “penetrated” his body ( *AFTA*, 82; Dodman 256). The doctor’s comment that “the foreign bodies were ugly, nasty, brutal. The Austrians were son of bitches” further indicates that his body had been penetrated by the enemy (82). Frederic’s bodily integration is hence undone not only by the trench mortar shell but also by the Austrians, the X-ray machine, and the Doctors; they represent the foreign Other that urge Frederic to reject his wounded knee.

From a different perspective, Frederic’s disgust towards his wounded knee can be understood as self-defense against his “disabled” condition. In *Consuming Gothic*, Lorna Piatti-Farnell notes that “disgust is deeply connected to notions of normality and appropriateness” (50). “Disgust,” Piatti-Farnell continues to write, “signals the breakaway point from what is proper and acceptable, from the very notion of safety and propriety” (51). Frederic’s regard of his wounded knee as Other can thus be interpreted as his attempt to perceive himself as able-bodied rather than recognize that he has become “disabled” or somehow deficient.

### “All to bits”

In “Going All to Pieces,” Trevor Dodman stresses that the traumatic violence in the novel lies in the fragmentation of human bodies caused by weapons of mass destruction (249-256). Throughout the novel, a great variety of war weapons are introduced, which include artillery ( *AFTA,* 158), rockets (47), big trench mortar shells (48), Skoda guns (47), grenades (106), rifles (106), pistols (130), naval guns (159), machine guns (162), iron shrapnel balls (162), cavalry (179), and so on. These destructive weapons blow Catherine’s ex-lover “all to bits” (17), injure Frederic’s knee, kill Passini, Aymo, and “one hundred and fifty thousand men on the Bainsizza plateau and on San Gabriele” and “forty thousand on the Carso” (116). Here, men’s bodies re spent in the war as if they hold little value: their bodies are fragmentized and their minds traumatized. This highlights industrial warfare’s fetishization of military technologies over human (living) bodies, leading to the massive production of destructive weapons and innumerable deaths.

In addition to military discourse, modern medical technologies (surgeries, x-rays) and doctors also play an essential role in creating fragmented disunity by imposing a mind-body dualism. As Emily Martin notes,

Many elements of modern medical science have been held to contribute to a fragmentation of the unity of the person. When science treats the person as a machine and assumes the body can be fixed by mechanical manipulations, it ignores, and it encourages us to ignore, other aspects of our selves, such as our emotions and our relations with other people.

(19-20)

In the novel, Frederic’s statement that “[d] octors did the things to you and then it was not your body anymore” reflects Martin’s critique above ( *AFTA,* 199). Another instance that mirrors Martin’s idea can be found in the episode that explains Rinaldi’s depression. “All summer and all fall I’ve operated. I work all the time,” as the military surgeon tells Frederic (146). Here, one understands that Rinaldi is forcefully turned into an anomaly, working like a machine. Moreover, he loses the human capacity for fundamental emotions: “I never think. No, by God, I don’t think; I operate” (147). This mechanization of Rinaldi reveals the military medical system as oppressive and inhuman. Frederic’s alienation of his wounded knee and Rinaldi’s repression represent a counter-narrative to industrial warfare that imposes technological fetishism.

## The *rotten* landscapes

### “This is the picturesque front”

The landscape of rotted and dead bodies represents another storied matter that showcases the horror of war. Unlike humans, who can speak for themselves, the natural world often remains a voiceless victim of war. Nevertheless, in *A Farewell to Arms*, a layer of non-anthropocentric consciousness resurfaces through the narrator’s descriptions, making the voices of nonhuman entities visible. Frederic’s non-anthropocentric consciousness is noticeable from the beginning of the novel. While observing Gorizia, Frederic states,

The river ran behind us and the town had been captured very handsomely but the mountains beyond it could not be taken and I was very glad the Austrians seemed to want to come back to the town some time, if the war should end, because they did not bombard it to destroy it but only a little in a military way.

( *AFTA,* 5)

Although the army has transformed Gorizia into a military town that includes hospitals, artillery, cafes, the shell-marked iron of the railway bridge, the smashed tunnel by the river, broken houses with plaster and rubble in the gardens, Frederic claims that he is “very glad” only because the town has not been ruined completely. ( *AFTA,* 5-6). Here, the sense of happiness embedded in Frederic’s description shows his hope for the end of the war (“if the war should end”) and his relief that not all-natural landscapes, especially “the mountains,” have been transformed into battlefields ( *AFTA*, 5).

In the novel, a counter-narrative on human-centrism can be found in Frederic’s association between the human body and the natural landscape. While observing Gorizia, Frederic claims, “the forest had been green in the summer when we had come into the town but now there were the stumps and the broken trunks and the ground torn up” ( *AFTA,* 6). Here, as Victoria Addis (2018) points out, phrases such as “stumps” and “broken trunks” recall “the male body, and more specifically, with the injuries and amputations that resulted from fighting in the war.” This linking of human bodies to nonhuman entities equalizes the subjectivity and agency ^8^ between the two, showing that both human and nonhuman *bodies* are victims of industrial warfare. According to anthropologist Joseph P. Hupy, the result of this first industrial-scale mechanized warfare is so devastating in terms of human loss of life and environmental cost that the war is regarded as an “anthropogenic disturbance” (418). In the novel, this anthropocentric destruction is committed by combatants who “marched as though they were six months gone with child” (4). Here, as Aime L. Pozorski notes, the phrase “to be gone with child” means “to be pregnant” (79). This linking of soldiers’ arms to maternal arms serves as an ironic representation that indicates the murder of children by the military arms. Frederic’s juxtaposition of soldiers’ arms and the maternal arms thus implies the front as a field of sterility. Historically, the French writer Henri Barbusse who fought on the Western Front also identified the battlegrounds as “‘fields of sterility’ where ‘frightful loads of dead and wounded men alter the shape of the plain’ and ‘everything appears turned over … full of rottenness and smelling of disaster’” (qtd. in Keller). As Joseph P. Hupy in “The Environmental Footprint of War” also notes, the environmental consequences of The Great War “obliterated forests” and “significantly altered the landscape” beyond recognition, creating “wide swathes of destruction, limited only by the range of artillery shells” (412). “Perhaps the best-known example of this swath of destruction,” Hupy continues to claim, “is the Western Front, a sinuous line of trenches craters, bunkers, and barbed wire averaging 20 km in width and stretching from the English Channel to the border of Switzerland” (413). Indeed, when Frederic looks at Biansizza after the war, he laments, “I had not realized it was so broken up” (159). This description recalls Barbusse’s claim that “‘[w] here there are not dead, the earth itself is corpselike”’ (qtd. in Keller). Frederic’s referring to the battlefield as “the picturesque front” thus indicates the wounded and dead human bodies and the broken, rotten, corpselike landscapes destroyed by modern destructive weapons (17).

### “I did not have the feeling that it was really over”

In the novel, Frederic’s frustration toward the anthropocentric destruction further increases when he sees that Caporetto has shifted from a charming “white” and “clean little town” to a landscape of death and chaos with “many sick” ( *AFTA,* 144). ^9^ This enhances his desire to escape toward the undisturbed land to “forget the war” and to have “a separate peace” (211). As he states, “I wanted to go to Austria without war. I wanted to go to the Black Forest. I wanted to go to the Hartz Mountains” (31).

Frederic’s impulse to escape the battlefields is linked to his witnessing the destruction of those towns. From the collapse of the older townscape to replacing the new landscape with fragmented and dead bodies, the whole process is mapped and set out as a visual geography of helplessness through Frederic’s eyewitness account. Here, the act of viewing is a “condition of passivity,” a form of violence one “can neither flee from nor defend against” (Nayar, 30). Furthermore, “[w] hat the eyewitness documents is a set of effects—primarily horror—and nothing beyond it. There is nothing intelligible about the events they see […] but what they apprehend is brutal, irreducible dying” (Nayar, 43). This, twinned with the fact that he is part of the destruction, makes Frederic feel extraordinarily helpless. Even after he flees the war with Catherine, this sense of helplessness continues to dominate him, making him think that he can never escape war: “I did not have the feeling that it was really over” ( *AFTA,* 213). Here, Frederic’s case symbolizes not an individual but a collective post-war experience. All combatants and citizens who witness the landscapes of dead and dying bodies experience the collapse of recognition and intelligibility. The process of gazing at the dying landscapes and dead bodies on the battlefield strengthens the sense of helplessness through collapsing what is perceived as “normal” in oneself, confusing one’s recognition of the world. In other words, the documentation of an *unmaking* of the world leads to the unmaking of subjectivity, resulting in the formation of helplessness. From this respect, the monstrosity of war lies in the collapse of subjectivity caused by the forced view of the landscapes of dying and dead bodies.

## Gothicized rain

Apart from food, bodies, and the landscapes, rain in the novel represents another storied matter worth exploring. In the novel, rain often creates a somewhat gothic atmosphere that is cold, dark, and chaotic and reflects and intensifies the feelings of helplessness, uncanniness, fear, and depression within the characters. This gothicized quality of rain can be found in the very first chapter of the novel, whereby rain corresponds directly with the coming of cholera and death: “[a] t the start of the winter came the permanent rain and with the rain came cholera” ( *AFTA,* 4). Moreover, as referenced earlier, during their idyllic time in Milan, Catherine reveals to Frederic her morbid reaction to rain. Furthermore, the Italian retreat from Caporetto is covered by dark and cold rain: “[a] s we moved out through the town it was empty in the rain and the dark except for columns of troops and guns that were going through the main street” (169). During the retreat, Aymo is shot to death in the rainy mud. “He looked dead. It was raining,” as Frederic claims (185). Later, when Catherine is having a Caesarean section at the hospital, it starts raining. At last, after bidding farewell to his dead wife, Frederic walks back from the hospital to the hotel “in the rain” (284).

The association of rain with disease, death, and war leads Malcolm Cowley to call the rain “a conscious symbol of disaster” (16). Similarly, Philip Young regards the rain as a metaphor for death ( 1966: 88). Furthermore, Carlos Barker associates rain with suffering, war, death, irreligion, and low-lying plains, putting them into the “not-home” category; at the same time, elements such as mountains, health, happiness, good-life, and God belong to the concept of “home” ( 1972: 102). ^10^ Thus, rain serves atmospheric and symbolic purposes in the novel. It is possible to argue that rain serves as a gothic “actant” ^11^ that plots human relationships and alters the course of events in the novel. By emphasizing the destructive-creative agency of the rain (accompanied by snow and wind) in the novel, the notion of human domination over nature is called into question, suggesting an ecocritical awareness to challenge the anthropocentric perceptions that encourage the spread of war.

The destructive-creative agency of rain is clear from the beginning of the novel. Despite the coming of cholera when the permanent rain comes in winter, increasing sick and dead people, Frederic points out, “in the end only seven thousand died of it in the army ( *AFTA,* 4; italics added). The word “only” emphasizes that the number of deaths from the war is far more significant than those who died from cholera. This highlights the destructive impacts of warfare on humanity compared to that of the winter rain. In fact, the agency of rain is viewed as “destructive” only because it is not beneficial for the people at this point. However, the winter snow stops the war in the novel: “There will be no more offensive now that the snow has come” (7). This then allows Frederic to take a personal adventure on his own: “I went everywhere. Milan, Florence, Rome, Naples, Villa San Giovanni, Messina, Taormina” (10). When Frederic returns to the front in the spring, he sees that “the fields were green and there were small green shoots on the vines” (9). Thus, from a non-anthropocentric standpoint, the winter rainfall and the snow are significant for the revival of life in nature. In “Winter Precipitation and Forests,” it is stated that the “infiltration of water into the ground occurs most efficiently during times when the forest is dormant.” The rainwater and melted snow offer a slow and steady flow of water into the forest soil so that life will emerge again as the spring begins. In this sense, the winter rain and snow propel (non) human things into “new relationships and new material embodiments” (Duckert, 115).

Another instance that shows the rain’s destructive-creative agency can be found in the episode of the Caporetto retreat, which Frederic describes as “wet and sullen” ( *AFTA,* 163). Historically, the Italian armies did their best to contain the German assault under torrential rain and freezing fog during the retreat. As colonel Francesco Pisani noted, “[t] here was total confusion, the road was almost entirely blocked by a mass of troops, carts, horses, trucks, artillery pieces, mules, and supplies … in the chaos, the freezing fog and the rain” (Wilcox, para. 7). In the novel, a similar situation is shown in Frederic’s description that “along the crowded roads we passed troops marching *under the rain*, guns, horses pulling wagons, mules, motor trucks, all moving away from the front. There was no more disorder than in an advance” (163-64; italics added). Here, rain’s destructive agency is reflected in its intensification of the disorder, the chaotic situation existing alongside the feelings of horror and helplessness among the people. Interestingly, the incident of the Caporetto retreat also allows for the life-giving quality of rain. As Frederic notes, “I was certain that if the rain should stop and planes come over and get to work on that column that would all be over. All that was needed was for a few men to leave their trucks or a few horses to be killed to tie up completely the movement on the road” (173). With the rain, the retreat is plunged into chaos. However, the situation would have been even worse without the rain as more soldiers would have been wounded and killed. In this respect, rain “preserves life” (Rennie, 38).

The fact that rain (and snow) is attributed to both the destruction and preservation of life foreshadows its destructive-creative agency, indicating that rain exerts a subjectivity of its own, a type of existence indifferent to humanity. When it rains, it manifests “‘ *Thing-Power:* the curious ability of inanimate things to animate, to act, to produce effects dramatic and strange” (qtd. in Duckert, 115). Rain’s ability to make things happen leads to the statement that “rain is not merely a metaphor *for* life, it is lively and *a* life, life defined in her own words” (Duckert, 115). In *Vibrant Matter*, Bennett also asks, “Does life only make sense as one side of a life-matter binary, or is there such a thing, a mineral or metallic life, or a life of the it in ‘it rains’?” (53). The fact that Hemingway, who was “depressed by rain” and “frequently complained about it,” constructed his novel heavily upon the agencies of rain seems to propose that rain is, indeed, a *vibrant* matter (qtd. in Grissom, 108).

### “There is no such thing as getting above the rain”

Rain’s destructive-creative agency, together with its random, wild, and unsympathetic qualities, emphasizes the fact that “there is no such thing as getting above the rain” (Cathcart, 95). Indeed, rain appears not only in the realm of reality but also in the world of fantasy. While taking a short break during the retreat, Frederic falls asleep and dreams of Catherine being brought to him by the western wind and rain:

Blow, blow, ye western wind. Well, it blew and it wasn’t the small rain but big rain down that rained. It rained all night. You knew it rained down that rained. Look at it. Christ, that my love were in my arms and I in my bed again. That my love Catherine. That my sweet love Catherine down might rain. Blow her again to me.

( *AFTA,* 171-72)

In Frederic’s dream, the association between Catherine and rain is once more emphasized. However, rain is romanticized this time: “That my sweet love Catherine down might rain” (172). As Tait Keller points out, “[t] he war’s impact on the land horrified university-educated soldiers groomed in the romantic appreciation for nature.” Even though the romanticization of the rain is itself an anthropocentric perception, it still overturns the idea of nature as a passive background, a view strengthened by the development of industrialization and mechanistic science (Merchant, 293, 294). In Frederic’s dream, the romanticized rain and wind are variable, creative, and lively. They save Frederic by blowing his lover to him and hence, *blowing* away the horror and pressure of being in war. Frederic’s romanticization of the rain and wind can thus be seen as his emotional resistance against the cruel, cold, mechanic war.

In the novel, Frederic’s mental resistance against the monstrosity of war is further reflected in his juxtaposition of the stormy weather and the weapons of war. Standing at the front line the day before the retreat begins, Frederic notes, “[i] t stormed all that day. The wind drove down the rain and everywhere there was standing water and mud” ( *AFTA,* 161). Under this stormy weather, Frederic and the others fight “in the dark in the rain” (161). According to Frederic, “[t] here were much shelling and many rockets in the rain and machine-gun and rifle all along the line … and between the gusts of wind and rain we could hear the sound of a great bombardment far to the north” (162). Here, there are two kinds of war going on. While the storm is a nonhuman disturbance generated by wind and rain, the battle is an anthropocentric assault marked by machine guns, rifles, smoke balls, and bombardments. However, these forces possess a different nature and impact. Firstly, rain is indifferent, while guns and bullets, on the other hand, are often used to fulfill egoistic delusions of domination. Secondly, rain’s autonomous forces resist drawing “the separations between climate and culture, life and matter, and subject and object” (Duckert, 116). In the novel, rain not only blurs the boundary between fantasy and reality, allowing Frederic to reconnect with Catherine but also keeps on living its life: “It rained all night” (171). In contrast, war weapons are objects used to objectify, disconnect, and eradicate all forms of life. In this respect, Frederic’s juxtaposition of the stormy weather with war weapons seems to propose a non-anthropocentric view that highlights the egoistic nature of warfare.

### “I … walked back to the hotel in the rain”

Toward the end of the novel, rain further points toward the naturalist philosophy of human existence. After Catherine dies, Frederic leaves the hospital and walks back to the hotel “in the rain” ( *AFTA,* 284). This recalls Catherine’s line, whereby she sees herself dead in the rain: “I’m afraid of the rain because sometimes I see me dead in it” (110). Nevertheless, there’s nothing intrinsically evil about the rain as Catherine’s hemorrhages occur randomly: “It’s just nature giving her hell” (274). After all, as Frederic comes to realize, whether, in war or love, his influence upon the outcome of events is insignificant. ^12^ Eventually, the universe kills indifferently. “You died. You did not know what it was about. You never had time to learn. They … killed you gratuitously like Aymo. Or gave you syphilis like Rinaldi. But they killed you in the end. You could count on that. Stay around and they would kill you” (280). Here, Frederic’s use of the term “they” reveals his realization of one’s place in the more-than-human (their) world. More specifically, human agency is conditioned by the natural laws of cause and effect beyond humans, and nobody can transcend these forces.

Just like one cannot stop the rain from falling, the death of Catherine will not stop the world from moving forward. “Things happen all the time. Everything blunts and the world keeps on … It never stops” ( *AFTA,* “Appendix II” 312). As for Frederic, who is still alive, he understands that he must move forward in his life: “[t] he rest goes on and you go on with it” (312). However, in defiance, Frederic also writes, “you have to stop a story. You stop it at the end of whatever it was you were writing about” (312). Following this, the ending scene, whereby Frederic leaves the hospital and walks back to the hotel “in the rain,” embodies two layers of implication (284). On the one hand, it can be interpreted as Frederic’s attempt to move forward in his life, as stopping the story here involves moving on rather like the indifferent rain. On the other hand, the scene also reveals a difficulty to move on, as Frederic ends the story in a rather sudden, silent, and abrupt ^13^ way, which might imply his inability to process his grief. However, ironically, since the living memory of Catherine is associated with rain, the only way not to forget about Catherine is, hence, to remain in the rain. Following this, while it seems to rain on Frederic’s behalf, ^14^ it also rains indifferently. In fact, it simply rains when it rains.

## Conclusion

*A Farewell to Arms* is a novel about war, love, and trauma. But more than that, it is also a novel about material bodies. The rotten food, fragmented human bodies, broken landscapes, and the gothic rain—all these are storied matters. Yet, if we look closely enough, we can discover their narratives. By digging into the rotten, uncanny, and gothic representations of the food, bodies, landscapes, and rain in the novel, one comes to understand the absurd, dualistic, destructive, and cannibalistic aspects of industrial warfare. These posthuman narratives thus counteract the discourses of military and modern medical science, suggesting that warfare is the ugliest, darkest, and most disturbing aspect of humanity.

The suggestion that warfare is a grotesque form of cannibalism is emphasized in the “rotten food” chapter, whereby eating rotten food is juxtaposed with killing/cannibalizing human living bodies on the Western front. Moreover, if one sees the symbolic connection between the phrase “cooked” and “traumatized,” one understands that the four-year-long war represents a slow cook/traumatizing process that transforms one into both the eater (traumatizer) and the eaten (victim). Meanwhile, soldiers on the Western front are encouraged to fight for the absurd value of heroism and the glory of war without even filling their stomachs. It is the corrupted food distribution system in the army that causes starvation and death among these soldiers. Realizing this, Frederic cannot help but lament the absurdity of war that leads to the meaninglessness of human sacrifice.

Another monstrous characteristic of warfare lies in its objectification and fragmentation of living human bodies, which can be found in the episode of Frederic’s confrontation with his injured body part. In the novel, Frederic’s repudiation of his left knee is deeply associated with the objectification of his body by the doctors and the x-ray machine. Together with the foreign object inserted into his knee, these actions painfully penetrate Frederic and undo his bodily integrity. Frederic’s rejection of his knee is thus a self-defensive act. Another instance is shown in the case of Rinaldi, where the military surgeon is forced to work like a machine for months, causing him to suffer from mental health problems. Industrial warfare’s fetishization of medical/military technology over the living human body can be observed through these cases. Indeed, a variety of weapons are created for the sole purpose of destroying and killing. As a result, countless lives—including Catherine’s ex-lover—are blown “into bits” by weapons of war. In this sense, the fragmented bodies question the violent and violating objectification of living human beings by medical science and military technology.

War’s fondness for military technology further encourages the destruction of natural landscapes. Through industrial war, battlegrounds and towns are transformed into landscapes of wounded and dead bodies. Having witnessed this “picturesque” ( *AFTA*, 17) transformation, Frederic feels frustrated as he is responsible for causing this anthropogenic disturbance *.* At the same time, he is also a victim based on his position as a passive, helpless viewer. Frederic then laments the ecological disaster caused by the war in his juxtaposition of the wounded human bodies with the broken landscapes. Here, Frederic’s anti-war consciousness shows that warfare is a destructive force that annihilates the subjectivity and agency of (non) human beings.

The dynamic narrative of the rain also plays a crucial role in revealing Frederic’s non-anthropocentric consciousness. In the novel, rain is a force that dominates human relationships and catalyzes events. While rain’s destructive agency, represented by the gothic rain in the novel, tends to destroy lives, its creative agency, expressed by the romanticized rain, reconnects relationships and preserves lives. After all, rain’s unpredictable, uncontrollable, and indifferent nature reveals that it holds a subjectivity of its own. In the novel, Frederic's promotion of this non-anthropocentric agency represents a counter-narrative to the human-centric war. Moreover, the connection between rain and Catherine’s sudden death urges one to acknowledge our place in this world. Humans are not as significant, influential, and independent as we assume; in fact, we are conditioned by indifferent forces and laws of nature. This conception is once again emphasized in the ending of the novel, where the rain seems to cry on Frederic’s behalf, but the fact is that it simply rains because it can. This points out the perception that the natural world is passive and feminized as human-centric and biased.

Suppose we regard *A Farewell to Arms* as an ecosystem. In that case, this essay serves to demonstrate how industrial war has knocked the novel-as-ecosystem intensely out of balance and turned it into something distinctly “rotten.” Hence, an eco-gothic emphasis of the agentic roles played by the rotten food, the uncanny bodies, the deformed landscapes, and the gothic rain in the novel reminds us that we derive much of what we recognize as our power, creativity, and accomplishments from the material things around us. This mode of thinking explains what kind of beings we are: we, humans, are inextricably entangled with our worldly surroundings, biologically, psychologically, culturally, and spiritually. This mode of thinking is essential in the Anthropocene, whereby human capacity has overloaded the Earth, resulting in uncontrollable environmental, social, cultural, and political problems. Only by shifting our perspective to a non-anthropocentric direction can we overcome these problems and reduce the incidents of warfare in the world. In this sense, an eco-gothic reading of *A Farewell to Arms* becomes vital as it not only shows the destructive impacts of war but also encourages a more fluid, realistic, and ethical reconstruction of humanity.

## Data availability

All data underlying the results are available as part of the article, and no additional source data are required.

## Footnotes

^1^
Drawing on *TATE*, the term “uncanny”—coined by the German psychiatrist Ernst Jentsch in *The Psychology of the Uncanny* in 1906—was referred to as “something new and unknown” that carries a negative implication. Sigmund Freud, however, repositioned the term in his essay *The Uncanny* (1919) as “the instance when something can be familiar and yet alien at the same time” (“Art Term: The Uncanny,” *TATE*). Uncanny, as Freud argued, was not just about the unknown but also “bringing out something that was hidden or repressed”; he called it “‘that class of frightening which leads back to what is known of old and long familiar’” (“Art Term: The Uncanny,” *TATE*).

^2^
According to *The Encyclopedia of Gothic*, the grotesque is “a protean form that joins tragic, trivial, and serious elements in such a way that it can be monstrous, absurd, humorous, and contradictory” (Hughes and Punter and Smith 307). Similar to the Gothic that explores the combination of beings (dead and alive, human and nonhuman), the grotesque concerns the transgression of boundaries (for instance, between normality and abnormality). Both the Gothic and the grotesque are related to Julia Kristeva’s “abject” (“‘the in-between, the ambiguous, the composite’”), a concept dealing with “a disturbing identity that disrupts order and the given system” (qtd. in Hughes and Punter and Smith 309). The grotesque is also associated with the uncanny in the sense that they both deform what is familiar. The standard definition of the term “grotesque” is “‘[d] istorted, incongruous, or fantastically ugly in appearance or style; bizarre; outlandish’” ( Sullivan 1996: 60). The term also carries the connotation of deformity and disability. After World War I, the prosthetic industry increased in a remarkable way which inspired the Dadaists to imagine a grotesque race of half-mechanical men (Dickerman 3-4). Alternatively, Ernst Friedrich, the founder of the Berlin Peace Museum and the author of *War Against Peace* (1924), used grotesque photographs of mutilated victims of World War I as a medium to promote peace.

^3^
Ecological Gothic is an innovative research field led by Andrew Smith, William Hughes, Monika Elbert and others (Silviya and Immanuel 25). Notable publications regarding the research include Smith and Hughes’ edited collection *EcoGothic* (2013), a special issue of the journal Gothic Studies entitled “Introduction: The EcoGothic in the Long Nineteenth Century” (2014) by David Del Principe, and Dr. Pramod K. Nayar’s recent work, *Bhopal’s Ecological Gothic* (2017).

^4^
Ford Maddox Ford, who wrote the first introduction to *A Farewell to Arms*, also claims, “Hemingway’s words strike you, each one, as if they were pebbles fetched fresh from a brook. They live and shine, each in its place” (qtd. in *AFTA* “Introduction” XV).

^5^
I use the term “storied” to emphasize the agency and the narrative ability of nonhuman matter. In “Posthuman Environs,” Jeffrey Jerome Cohen draws on Serpil Oppermann and Serenella’s Iovino’s claim that “all matter is *storied*” and asks that “[m] ight ‘storied matter’ pulse in fundamental units—nouns, verbs, syllables, morphemes?”; “Might matter be inscribing us, rendering humans the record of a Disanthropoce that unfolds regardless of what epochs we declare?” (25). The term “storied” matter is thus a counter-narrative against the anthropocentric view that regards nonhuman matter as passive and lacking subjectivity.

^6^
Traumatic experiences that involve the development of altered states of consciousness regarding “the sense of body ownership and agency” as well as “depersonalization (where parts of the body or the entire body itself is perceived as detached and out of control)” may lead to post traumatic stress disorder (PTSD) (Rabellino *et al*. “Abstract”).

^7^
According to the *National Health Service*, common PTSD symptoms include, but are not limited to trauma re-experiencing (via nightmare, flashback, physical sensation), avoidance and emotional numbing, hyperarousal (anxious and difficulty to relax and concentrate), mental problems (depression, phobias), destructive behavior (drug or alcohol misuse), physical symptoms (headache, dizziness, stomachache, chest pain).

^8^
Agency, as Stacy Alaimo emphasizes, is a performativity that “allows matter its due as an active participant in the world’s becoming, in its ongoing ‘intra-activity’” ( 2008, 248). “Agency,” more specifically, in Karen Baran’s words, “is not aligned with human intentionality or subjectivity”; it is a “‘doing’/ ‘being’ in its intra-activity”; it is “the enactment of interactive changes to particular practices through the dynamics of intra-activity” (2003, 827). Intra-action is a neologism created to emphasize “ *the mutual constitution of entangled agencies.* That is, in contrast to the usual ‘interaction,’ which assumes that there are separate individual agencies that precede their interaction, the notion of intra-action recognizes that distinct agencies do not precede, but rather emerge through, their intra-action. It is important to note that the ‘distinct’ agencies are only distinct in a relational, not an absolute, sense, that is, *agencies are only distinct in relation to their mutual entanglement; they don’t exist as individual elements*” (Barad, 2007, 33).

^9^
Historically, Colonel Francesco Pisani, who was ordered to Caporetto on 23 October 1917, also wrote in his official post-battle report that Caporetto was in a total mess, whereby the entire road was blocked by a mass of troops, trucks, artillery pieces as well as “the wounded […] who had been abandoned in the road” ( Wilcox, 2017). Overall, the Italian army had forty thousand killed or wounded soldiers, followed by the two hundred and sixty-five thousands of prisoners and dispersed soldiers during the Caporetto retreat ( Wilcox, 2017). Since then, the word “caporetto” functions as “a metaphoric expression for a contest where the defeated party is so thoroughly outplayed, outmaneuvered, and overpowered that it can do little more than dissolve into confusion and chaos” ( Debeljak, 2015).

^10^
According to Carlos Baker, *A Farewell to Arms* is organized connotatively around two poles: “the concepts of Home and Not-Home” (101). Baker links the Home-concept to “the mountains; with dry-cold weather; with peace and quiet; with love, dignity, health, happiness, and the good life; and with worship or at least the consciousness of God” (102). On the other hand, the Not-Home concept is connected to low-lying plains; with rain and fog; with obscenity, indignity, disease, suffering, nervousness, war and death; and with irreligion” (Baker 102).

^11^
An “actant,” as Jane Bennet notes, is “a source of action that can be either human or nonhuman; it is that which has efficacy, can do things, has sufficient coherence to make a difference, produce effects, alter the course of events” (viii). Rain is thus a type of distributed agency that outstrips the rational, intentional agent of humanism.

^12^
As Mary Prescott also points out, “Frederic’s sense of his lack of control and power in a world relentlessly indifferent to human suffering” is deeply linked to “the rain,” which is in association with the war, his trauma, and Catherine’s hemorrhages (45-6).

^13^
As F. Scott Fitzgerald also wrote, “[w] hy not end the book with that wonderful paragraph on p. 241 [pp. 317-318 in print]. It is the most eloquent in the book and would end it rather gently and well” (qtd. in Oldsey 495).

^14^
In his article, Kenneth Burke sees the raining scene as a reflection of Frederic’s inaudible cry. As he asks, “‘do you not find the very heavens are weeping on his behalf?’” (qtd. in Oldsey 499). To support his statement, he quotes Verlaine’s line “‘It rains in my heart as it rains on the town’” (qtd. in Oldsey 499).
