# Supplementary material for: The “Rotten” matter in A Farewell to Arms: An Ecological Gothic reading
Source: F1000Res. 2022 Nov 23;10:1287. Originally published 2021 Dec 15. [Version 2] doi: 10.12688/f1000research.75482.2 (PMC10466005; doi:10.12688/f1000research.75482.2)
Supplement: Peer review history

# Peer review: Reviewer response for version 2

**DOI:** 10.5256/f1000research.141223.r198561

**Published:** 2023-08-29

**Authors:** Carol Senf

**Affiliations:** 1 School of Literature, Media, and Communication, Georgia Institute of Technology, Atlanta, Georgia, USA

The essay takes an eco-gothic perspective to explore Hemingway's *A Farewell to Arms *and argues persuasively that the novel can be read as a condemnation of technological warfare.

 It concentrates on rottenness in the novel -- including rotten food, rotten bodies, rotten landscapes, and then concludes with a section on Gothicized rain. The section that focuses on rain is compelling, but the link between the rottenness in the earlier sections might be clearer. I wonder, in fact, whether the essay would be more coherent if it were reorganized to begin with the section on Gothicized rain, which is an overarching trope throughout the novel.

 The central argument would also be stronger if material from footnotes (especially 1, 2, 3, and 8) were integrated into the essay itself. Reserve footnotes for information that is not essential to the main argument (e.g. 4, 6, and 7)

 The writer needs to provide full source data. In fact, the writer (or someone else) has already indicated sources that are incomplete.

Is the work clearly and accurately presented and does it cite the current literature?

Yes

If applicable, is the statistical analysis and its interpretation appropriate?

Not applicable

Are all the source data underlying the results available to ensure full reproducibility?

Partly

Is the study design appropriate and is the work technically sound?

Yes

Are the conclusions drawn adequately supported by the results?

Yes

Are sufficient details of methods and analysis provided to allow replication by others?

Yes

Reviewer Expertise:

The Long Nineteenth Century, the Gothic.

I confirm that I have read this submission and believe that I have an appropriate level of expertise to confirm that it is of an acceptable scientific standard, however I have significant reservations, as outlined above.

# Peer review: Reviewer response for version 1

**DOI:** 10.5256/f1000research.79360.r116255

**Published:** 2022-08-04

**Authors:** Hideo Yanagisawa

**Affiliations:** 1 Meijo University, Nagoya, Japan

This paper is an attempt to reread Hemingway's *A Farewell to Arms* as a counter-narrative to anthropocentrism, using "eco-gothic," the latest research method in environmental literary criticism. The originality of the attempt itself and its challenging attitude should be highly evaluated. However, I would like to point out two problems.

1. In the chapter "The rotten food", the author focuses on the food shortage during the war and points out the contradiction of civilized society that makes even the very primitive act of "eating" unfeasible. On the other hand, I find it contradictory that the author points out "war = barbarism" and "civilization = healthy food control" while pointing out in Frederic’s linking of fresh food to civilization from Chapter 34. In other words, the author should consider the fact that war itself is inseparably linked to civilization.
2. In the chapter "Gothicized Rain," the author interprets Frederick's romanticization of rain to mean the restoration of people's romantic view of nature destroyed by war, and that this is Frederick's criticism of the anthropocentrism symbolized by war. However, the contradiction that the romantic view of nature itself is anthropocentric needs to be resolved.

Is the work clearly and accurately presented and does it cite the current literature?

Yes

If applicable, is the statistical analysis and its interpretation appropriate?

Not applicable

Are all the source data underlying the results available to ensure full reproducibility?

Yes

Is the study design appropriate and is the work technically sound?

Partly

Are the conclusions drawn adequately supported by the results?

Yes

Are sufficient details of methods and analysis provided to allow replication by others?

Yes

Reviewer Expertise:

Ernest Hemingway, American literature, English-language, Representational Culture

I confirm that I have read this submission and believe that I have an appropriate level of expertise to confirm that it is of an acceptable scientific standard, however I have significant reservations, as outlined above.

# Peer review: Reviewer response for version 1

**DOI:** 10.5256/f1000research.79360.r128703

**Published:** 2022-03-30

**Authors:** Scott Slovic

**Affiliations:** 1 University of Idaho, Moscow, ID, USA

This article is an exceptionally imaginative and convincing representation of the eco-gothic aspects of Hemingway’s famous novel. I am especially impressed by the way Lay Sion Ng draws parallels between the human and environmental facets of the narrative and between the violence of war and the unsettling aspects of normal human life, including the violence and mortal decay associated with eating and preparing food. The essay relies heavily on this strategy of establishing unexpected and jarring parallels, and this appears to be one of the goals of the author’s application of material ecocritical and eco-gothic methodologies and conceptual paradigms. But I find myself wondering whether establishing such parallels should be an end in itself or whether there might be additional, ulterior purposes for such a study.

 I see that one of the scholarly sources in this article, mentioned briefly in a footnote, is Pramod K. Nayar’s 2017 book *Bhopal’s Ecological Gothic: Disaster, Precarity, and the Biopolitical Uncanny*. Nayar’s study of the literature and art that have emerged following the 1984 industrial disaster in southern India also places heavy emphasis on surprising and unsettling connections and parallels. In “Conclusion: ‘Burial of an Unknown Child’ as Icon,” Nayar highlights the cognitive dissonance in this photograph by Raghu Rai, which the critic calls “gut wrenching.” The point of explaining this gut-wrenching application of gothic aesthetics to the representation of death and destruction caused by Union Carbide’s chemical spill in Bhopal is not merely to present an academic argument but to poignantly critique the social and environmental injustice associated with industrial activities. The exposure of children and other innocent people and organisms to deadly chemicals should be prevented—this is the moral core of Nayar’s work. At the outset of his book, in the Introduction,” he writes that “Literary-cultural studies embodying the ‘ethical turn’ have addressed human rights, democracy, torture and environmentalism in the past few years. Such studies enable us to see the rhetorical and discursive strategies employed in fields like Literature, films or comics that generate cultural models of victimhood, trauma, personhood, the Human, civilization or development” (xiv). I find that Professor Ng’s study does a very good job of illuminating the rhetorical and discursive strategies that Hemingway has intentionally or intuitively layered into his novel, but the ethical dimension of Hemingway’s eco-gothic “counter-narrative”—the anti-war critique—seems somewhat muted in this study and could perhaps be explained more directly and forcefully.

 Another key aspect of recent ecocritical work that connects the human body to the natural landscape—and particularly to the vulnerability and mortality of nature—is the effort to overcome the myth of human exceptionalism. In my own analysis of Wilfred Owen’s World War I poem “Dulce Et Decorum Est” in the *Routledge Handbook of Religion and Ecology* (2017), I argued that Owen’s graphic portrayal of the brutality of war and the physical suffering it entails is not merely a critique of war itself but also a puncturing of the “old lie” that “humans often tell themselves. Humans are not presented here as being exceptional, or separate, from the natural order of things, from living and dying. The vivid portrayal of injured and dying human beings in this poem demonstrates that we are mortal, that we are animals” (360). More recently, in *Ecocollapse Fiction and Cultures of Human Extinction* (2021), Sarah E. McFarland writes, “At issue … is how to reject the impulse of human exceptionalism that pervades Western thought and much speculative fiction by exploring those few texts that engage with the potential of human species extinction…. Becoming attuned to the inseparability of human and nonhuman worlds—what Donna Haraway calls ‘entanglement’—thus insubstantiates the exceptionalism experienced as part of the Western human tradition” (3). It seems to me that perhaps Professor Ng could do a little bit more in this study of Hemingway to place this discussion in the context of other contemporary ecocritical projects that break down the separation between the human and the nonhuman in order to critique on a fundamental psychological and philosophical level the idea of human exceptionalism. Further, and this goes back to Nayar’s work on Bhopal and his broadening of this project in the 2019 volume *Ecoprecarity: Vulnerable Lives in Literature and Culture*, the point of representing the vulnerability of human bodies and minds and human communities is to reinforce the precariousness of our own lives and the planet we depend upon for our existence.

 I find Professor Ng’s study to be well written and thoroughly researched. The article is very effective in presenting important narrative details in Hemingway’s novel and in demonstrating how these details—especially the breaking and rotting of human bodies and the natural environment—contribute to a deeply moving and unsettling eco-gothic aesthetic in *A Farewell to Arms*. It might be helpful, though, to explain more fully the ethical urgency of such an anti-war and anti-human-exceptionalist perspective, as expressed in Hemingway’s work and in a contemporary analysis of such a novel. Linking this article to Nayar’s work on Bhopal literature and culture and to McFarland’s review of “ecocollapse fiction” might be helpful.

Is the work clearly and accurately presented and does it cite the current literature?

Yes

If applicable, is the statistical analysis and its interpretation appropriate?

Not applicable

Are all the source data underlying the results available to ensure full reproducibility?

No source data required

Is the study design appropriate and is the work technically sound?

Yes

Are the conclusions drawn adequately supported by the results?

Yes

Are sufficient details of methods and analysis provided to allow replication by others?

Yes

Reviewer Expertise:

Ecocriticism, Interdisciplinary Environmental Humanities, American Literature, Comparative Literature, Environmental Communication

I confirm that I have read this submission and believe that I have an appropriate level of expertise to confirm that it is of an acceptable scientific standard.

## References

[1] : Ecocollapse Fiction and Cultures of Human Extinction. *London: Bloomsbury Academic* .2021;

[2] : Bhopal’s Ecological Gothic: Disaster, Precarity, and the Biopolitical Uncanny. *Lanham, MD: Lexington Books* .2017;

[3] : Ecoprecarity: Vulnerable Lives in Literature and Culture. *New York: Routledge* .2019;

[4] : Routledge Handbook of Religion and Ecology. *London: Routledge* .2017;355-363
